# Phase contrast imaging with inelastically scattered electrons from any layer of a thick specimen

**DOI:** 10.1016/j.ultramic.2022.113511

**Published:** 2022-07

**Authors:** Joshua L. Dickerson, Christopher J. Russo

**Affiliations:** MRC Laboratory of Molecular Biology, Francis Crick Avenue, Cambridge CB2 0QH, UK

**Keywords:** Inelastic phase contrast, CryoEM, Top bottom effect, *C*_c_ correction

## Abstract

A controversy exists as to whether the signal in a high resolution phase contrast electron micrograph of a particle in a thick specimen is the same irrespective of the particle’s position along the beam axis. Different conceptions of inelastic scattering and its effects on wave interference have led to radically different expectations about the degree of phase contrast vs. depth. Here we examine the information available from bright field phase contrast images of small crystalline particles on the top or bottom of a thick support. The support is an aluminium foil which has strong plasmon resonances that cause a large proportion of the electron beam to lose energy in transit. Phase contrast micrographs of the atomic lattice of two ensembles of platinum particles were measured in an energy loss window corresponding to the first plasmon resonance. The signal measured for particles on top was equal to that for particles on the bottom of the foil to within a 99% confidence interval, and the measurements exclude other models of depth dependent phase contrast in the literature to >5σ. These observations are consistent with quantum theory which considers dynamical effects as independent of event sequence and is distinct from the “top-bottom effect” observed in amplitude contrast. We thus confirm that phase contrast using inelastically scattered electrons can be obtained equally well from particles within any layer of a thick specimen.

## Highlights

• A controversy exists about phase contrast with inelastically scattered electrons.
• A definitive experiment is carried out to settle the debate.
• Particles on the top and bottom of a thick foil are imaged with inelastic scattering.
• Phase contrast can be obtained equally well from all layers of a thick specimen.

## 1 Introduction

The theory of inelastic scattering developed by Howie in 1963 [1] indicates that fast electrons which suffer energy loss to plasmon excitation in a material still contribute to Bragg reflections and the wave interference patterns found in images. This theory was further extended to include other forms of inelastic scattering [2] with careful reference to energy loss imaging experiments. The implications for the imaging of thick specimens were noted in these papers, albeit in the context of instrumentation that was limited in resolution relative to modern electron microscopes. As technology moved on and high resolution phase contrast imaging became a reality, Rose made it clear that inelastic scattering does not inevitably lead to a loss of coherence since the inelastically scattered wave interferes with the wave that has been elastically and inelastically scattered [3]. More recent holographic imaging with inelastically scattered electrons [4], [5] shows beyond a doubt that inelastically scattered electrons preserve both phase and amplitude information to atomic resolution. A particularly elegant explanation of coherence in elastic and inelastic scattering was provided again by Howie in 1979 [6], emphasising the point that the sequence of inelastic vs. elastic scattering has no appreciable impact on the degree of phase contrast in an image.

As the ranges of specimens amenable to electron imaging have expanded, both in the materials science and now biological communities, fundamental questions about image contrast once thought settled have re-emerged. So the question of whether phase contrast is uniform throughout a thick specimen has become a source of debate. Put another way: Does the sequence of elastic and inelastic scattering processes that occur as the electron transits the specimen influence phase contrast seen in the image? It might be tempting to take a limited view of electron specimen interactions that assumes the electron wavefunction is “collapsed” by an energy loss event, and thus treat the electron as a single particle emitted from the location of the event. This would mean that only particles towards the bottom of a thick specimen would contribute to inelastic phase contrast, as was assumed in a study of the role of inelastic scattering in phase-plate electron microscopy [7]. Conversely, one can take the view that an inelastic event following an elastic event preserves phase contrast but the reverse sequence does not [8]. This would mean only particles near the top of a thick specimen could contribute to phase contrast.

Empirical observations from experiments done using contrast formation mechanisms other than phase interference may have contributed to this confusion. Reimer showed that there is a clear and easily reproducible “top-bottom effect” when imaging a specimen under conditions in which phase contrast is absent and only amplitude contrast via incoherent scattering remains [9], [10]. In incoherent scanning transmission electron microscopy (STEM), this manifests as particles on top appearing sharper since the probe is broadened in transit [9]. Conversely in bright field transmission electron microscopy (TEM), this is seen as a blurring of particles on the top of a specimen that is several inelastic mean free path lengths thick [10]. Neither of these phenomena are a result of phase interference so theoretical treatments that include amplitude blurring effects as empirical terms in expressions for phase contrast [11], [12] do not obviously follow from these experiments in the incoherent scattering regime.

**Fig. 1:**
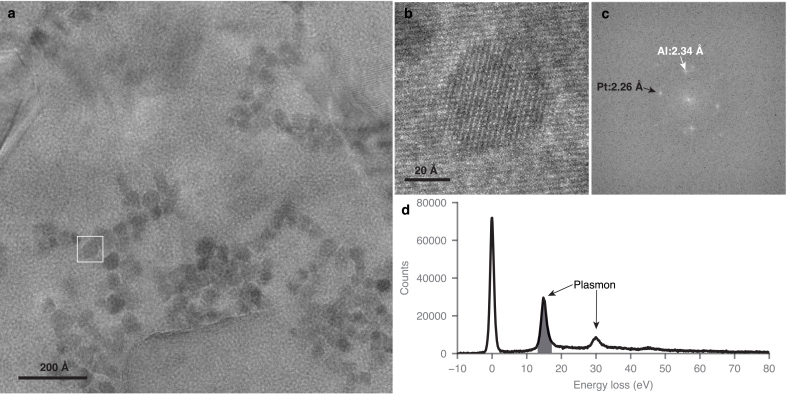
Platinum particles on *top* of an aluminium foil imaged with 15 eV energy loss electrons and a 300 keV primary energy. Panel (b) is an enlargement of the particle boxed in (a), and (c) shows the Fourier transform of (b), with arrows indicating the 111 reflections from platinum (black) and aluminium (white). An electron energy loss spectrum of the specimen in (a), showing plasmon peaks of aluminium at 15 and 30 eV. The energy range selected (15 ± 2 eV) for the imaging is indicated in grey.

Imaging particles with high resolution phase contrast at any depth in a thick specimen has renewed importance in the context of imaging biological molecules *in situ* [13]. Particularly in the case of chromatic aberration corrected imaging in thick specimens, which is considered more broadly in an accompanying paper [14], it is essential that the controversy described above is resolved. We have devised a simple experiment that should lay to rest any residual doubts about the theory of high resolution phase contrast vs. depth in images using inelastically scattered electrons. Specifically, we measure the power in the 111 reflection from an image of the atomic lattice of a small platinum particle on either the top or bottom surface of a thick aluminium foil. We collect only the electrons that have lost an energy commensurate with the plasmon resonance in the aluminium, which for the thickness of foil used, is ∼20% of the beam. If the sequence of elastic and inelastic scattering changed the amount of phase contrast, there would be a corresponding difference in the power from particles on top vs. on bottom. We also compare these measurements to simulations performed using the two models of depth dependent loss described above.

## 2 Materials and methods

### 2.1 Specimen preparation

Amorphous carbon foils were created using vacuum coating on mica as described in an accompanying paper [14]. The density of the carbon was measured by sedimentation in a chloroform/bromoform density gradient and was 1.7 g/cm${}^{3}$; the thickness was measured by AFM to be 660 Å. Suspended foils were then prepared by transferring the carbon onto the palladium side of 300 line per inch square mesh copper/palladium grids (Agar Scientific) by flotation on water [15]. Grids with the most complete carbon coverage were selected for use (Fig. S1). A 1100 Å thick layer of aluminium was deposited onto the foil side of these grids by electron beam evaporation at 9 kV using a custom electron beam evaporator (Moorfield) evacuated to a pressure of $1\times 10^{-7}$ mbar. The source metal was pellets of aluminium of purity 99.999% (Kurt J. Lesker). The thickness was measured during deposition using a calibrated crystal thickness monitor (Infinicon); the rate of deposition was 1 Å/s and the grids were nominally at room temperature (attached to a copper plate but not actively cooled) during the evaporation. The suspended foils were rendered hydrophilic by exposure to an Ar:O${}_{2}$ plasma mixture (9:1, using N6 grade source gasses, BOC) at 38.8 W forward power and 1.9 W reverse power in a commercial plasma chamber (Fischione 1070). The exposure time was 2 min. After plasma treatment, 0.5 μL of a solution of 50 Å diameter platinum particles (nanoComposix) suspended in water at a concentration of 1 mg/mL was pipetted onto one side of the grids (foil side) and the excess liquid was removed by blotting with filter paper (Whatman No. 1) from the same side. Inspection under a dissecting microscope during this process ensured that the particle solution did not cross to the other side of the grid or touch the tweezers. Platinum particles were chosen (as opposed to gold, lattice constant of 4.065 Å) since the lattice constant of platinum is different enough to that of aluminium (3.912 Å and 4.046 Å respectively) to make the 111 reflections easily distinguishable in the Fourier transform of the images (2.26 vs. 2.34 Å, see Fig. 1c).

### 2.2 Electron microscopy and spectroscopy

Images of platinum particles on an aluminium and carbon foil (Fig. 1a-c) were taken on a TEM (FEI Titan Krios G2) operating at a beam energy of 300 keV. The nominal magnification was set to 215,000×, corresponding to a magnified pixel size of 0.34 Å at the specimen, which was calibrated using the 111 reflection from aluminium at 2.34 Å. Data was collected using a BioQuantum electron energy loss imaging filter and K3 direct detection camera (Gatan) operating in counting mode with an exposure time of 4 s. The electron flux on the specimen was 15 e${}^{-}$/Å${}^{2}$/s and the total fluence in each 4 s image of the defocus series was 60 e${}^{-}$/Å${}^{2}$. The width of the energy filter slit was set to 4 eV and was centred on the 15 eV plasmon peak of aluminium (Fig. 1d). A defocus series was taken in 250 Å steps between −3500 and +3500 Å to ensure that the image series crossed the defocus value of minimum contrast. A SerialEM script was written to control the objective lens defocus during series acquisition [16]. Grids were clipped in clip rings with either the foil side down (facing the clip ring and towards the gun when loaded in the column) or the foil side up (towards the c-clip and away from the gun). Electron energy loss spectroscopy (EELS) was conducted to measure the position and width of the energy slit and ensure it was centred on the peak (Fig. 1d). A total of 38 defocus series were collected from the top and 27 series from the bottom type specimens. EELS was also conducted on a portion of the grids containing only carbon so that the thickness of the carbon foil after plasma etching could be measured (600 Å).

### 2.3 Data analysis and processing

Every image series contained 29 defocus values; each series was aligned using Unblur [17] to remove stage drift. The particles (including both sidebands) were selected and boxed out (250 × 250 px) for subsequent processing [18]. The intensity of the platinum 111 reflection at 2.26 Å resolution was measured from the Fourier transform of the particle image. For each defocus series, the image with maximal intensity in the reflection was taken as representative. This value was divided by the mean squared fluence, which takes account for changes in the number of electrons reaching the detector due to thickness variations across the specimen, and the mean was taken across all particles on a particular side.

### 2.4 Monte Carlo simulations

To compare our best understanding of the theoretical scenarios in the literature with the experimental results, we performed Monte Carlo simulations of particle scattering events under three scenarios. Neither the second nor the third scenarios are consistent with the well established theory of image formation described in the introduction [1], but we include them to try and capture the difference in contrast one might expect given the descriptions in Refs. [7], [8]. Phase contrast is generated in the image if and only if:

1. Each electron has a wavefront that accumulates elastically scattered information, and inelastic events can happen anywhere in the material.
2. The electron is elastically scattered only once in the particle and is not inelastically scattered thereafter.
3. The electron is elastically scattered only once in the particle, and is not inelastically scattered before this event.

Note in all three scenarios, the electrons must also have inelastically scattered to within the energy window corresponding to the filter (13–17 eV). The ratio of electrons from a ‘top’ type specimen to a ‘bottom’ type specimen under each scenario were then used for comparison with the experimental measurements described above.

For simplicity, the platinum particles were modelled as cylinders with a diameter of 50 Å and height of 33.5 Å, thus having the same volume as a 50 Å sphere. The total interaction cross section for each material was taken as the sum of the inelastic scattering cross section (calculated using a generalised oscillator strength model [19], [20]) and the elastic scattering cross section (taken from the NIST database [21]). The simulation was performed for $10^{7}$ electrons in each of the three scenarios; each scenario was run ten times for the particle on top and ten times for the particle on the bottom. The relative signal between the different scenarios was then calculated based on the ratio of electrons contributing to signal in each case (particles on top vs. bottom).

**Fig. 2:**
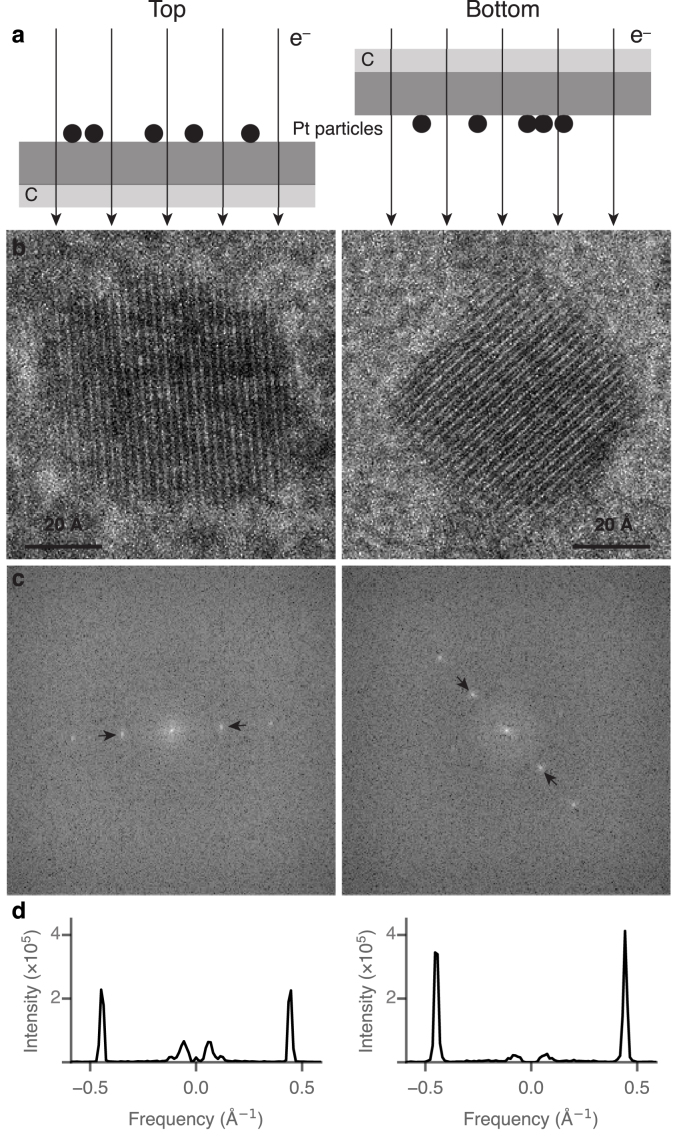
Phase contrast from particles on the *top* and *bottom* of an 1100 Å thick aluminium + 600 Å thick carbon foil. A diagram of the experiment is shown in row (a). Example phase contrast micrographs, taken under identical conditions at 15 eV energy loss and 300 keV primary energy, of platinum particles on the top and bottom are shown (b) with the power spectra of the cropped images (c) and a section through the power spectra in the directions indicated by the arrows (d). Note the low frequency peak was suppressed in (d) to aid in visualisation of the peaks.

## 3 Results & discussion

A representative energy filtered micrograph of platinum particles on top of an aluminium foil at 15 eV loss is shown in Fig. 1. The atomic lattice of the platinum particles and the aluminium polycrystalline foil are both clearly resolved. Whilst any individual platinum particle may have a slightly higher or lower intensity in the diffracted beams for several reasons – including size of the particle, angle with respect to the zone axis, the precise value of the defocus position and the local thickness and structure of the foil – over a large ensemble of measurements these variations are expected to average out. A total of 91 diffracting particles on top of the aluminium and 92 on the bottom were analysed. An example of each is shown in Fig. 2; the maximum intensity of the platinum 111 reflection across the defocus series was recorded for each particle and the mean value of the intensity from all the particles was then calculated. From these two particle sets, the mean normalised intensity in the diffracted beams from particles on top of the foil was $1.37\times 10^{6}\pm 9\times 10^{4}$ and the mean intensity for particles on the bottom was $1.37\times 10^{6}\pm 1.1\times 10^{5}$, where the units are counts and the error reported is the standard error in the mean. The standard deviations are $8.6\times 10^{5}$ and $1.06\times 10^{6}$ respectively. Using Student’s T-test, we can be confident that the two mean values are the same, with a p value of 0.99. To further compare this measurement to models of scattering where contrast is lost towards the top or bottom of the specimen, we performed Monte Carlo simulations for each case as described in Section 2.4. The simulations indicate that the power in the 111 reflection of platinum for top vs. bottom would have a ratio of 0.00650±0.00009 for scenario 2 in Section 2.4 and 99.3±1.3 for scenario 3. In comparison to the experiment (ratio of 1.00±0.10) both scenarios 2 & 3 are excluded to greater than a 5σ confidence level. We also note that in principle, electron channelling effects [22] could cause a top-bottom asymmetry in the phase contrast from crystalline specimens. But in the present case, the increase in angular spread due to the specimen is at least an order of magnitude smaller than is required to detect channelling effects in a 50 Å platinum particle and thus is negligible. Furthermore, electron channelling is completely absent in the amorphous biological specimens of interest.

It is clear from the results presented here that under the high resolution imaging conditions used in this experiment, there is no discernible difference in the inelastic *phase* contrast from particles on the top or bottom of a thick specimen, which is distinct from the top bottom effect for *amplitude* contrast. If one contemplates that there is only about one electron in the column at a time, this is reassuring. When considering the imaging of thick biological specimens using chromatic aberration correction, we can thus be confident that inelastically scattered electrons have the potential to contribute to phase contrast signal irrespective of where in the specimen the inelastic scattering event occurred.

## Declaration of Competing Interest

The authors declare that they have no known competing financial interests or personal relationships that could have appeared to influence the work reported in this paper.

## Appendix A Supplementary data

MMC S1 Figure demonstrating the measures taken to ensure that the particles are only present on one side of the specimen.
