# Supplementary material for: Phase contrast imaging with inelastically scattered electrons from any layer of a thick specimen
Source: Ultramicroscopy. 2022 Jul;237:None. doi: 10.1016/j.ultramic.2022.113511 (PMC9355894; doi:10.1016/j.ultramic.2022.113511)
Supplement: MMC S1 — Figure demonstrating the measures taken to ensure that the particles are only present on one side of the specimen. [file mmc1.pdf]

Supplementary information

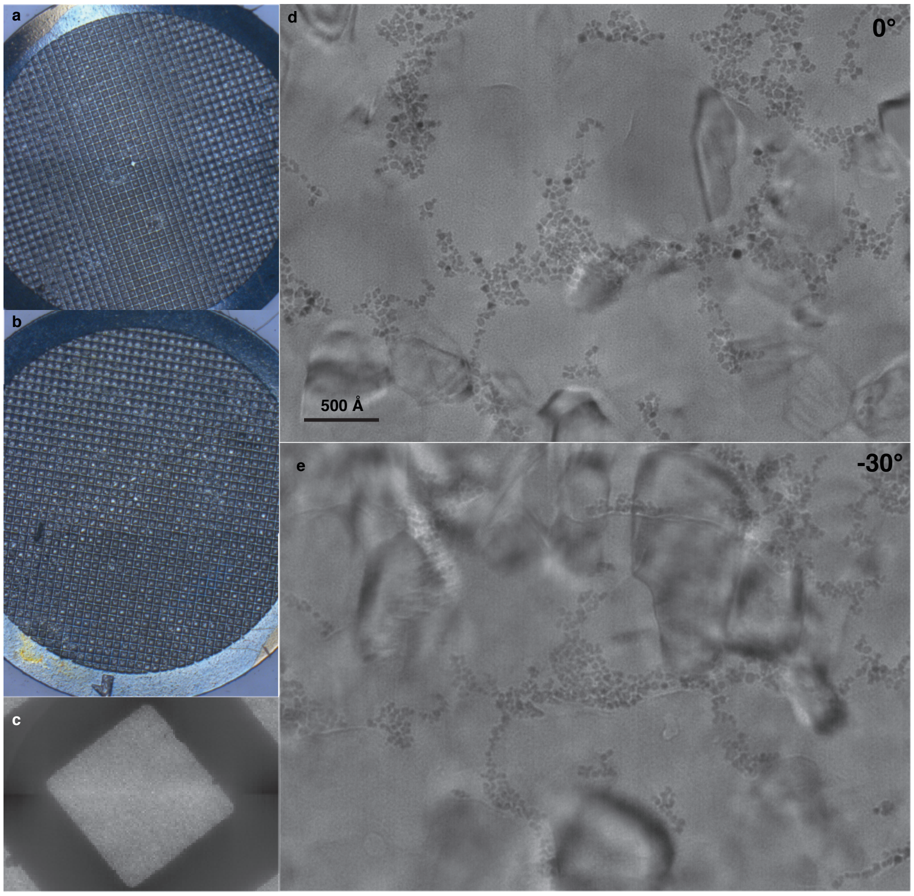

Figure S1: Images showing the steps taken to ensure that all particles are only on one side of the foil. Light microscope images in (a) and (b) show the selected specimens which have foils completely covering one side of the grid after deposition of the particles on one surface. Only intact squares were selected for imaging (shown in c). In addition, tilt pairs were collected at low magnification in the electron microscope at 0 and -30 degrees tilt (d & e), to verify the particles were all on one side. Two adjacent particles would be expected to move apart by $\sim$ 1000 Å if they were on opposite sides of the specimen; no particles moved in this way in the tilt pair.
